# A scalp to make you squirm: Cutaneous myiasis hiding a unique presentation of disseminated coccidioidomycosis

**DOI:** 10.1016/j.idcr.2025.e02417

**Published:** 2025-11-03

**Authors:** Jared Miguel Duldulao, Nina Modanlo, Sushma Boppana, Kristina Adachi, Huan Vinh Dong, Ishminder Kaur, Karin Nielsen-Saines, Jessica Terrell, Carol E. Cheng, Sanchi Malhotra

**Affiliations:** a University of California, Los Angeles, Department of Pediatrics, Pediatric Residency Program, Los Angeles, CA, USA; b University of California, Los Angeles, David Geffen School of Medicine, Los Angeles, CA, USA; c University of California, Los Angeles, Department of Pediatrics, Division of Infectious Diseases, Los Angeles, CA, USA; d University of California, Los Angeles, Department of Medicine, Division of Dermatology, Los Angeles, CA, USA

**Keywords:** Myiasis, Coccidioidomycosis, Scalp lesion, Fluorosis, Endemic mycoses

## Abstract

Coccidioidomycosis is predominantly a pulmonary disease, however dissemination can include unique cutaneous presentations. We report a previously healthy 11-year-old female with delayed diagnosis of cutaneous scalp coccidioidomycosis with disseminated disease with secondary wound myiasis and bacterial superinfection. She required extensive surgical debridement, eventually requiring a skin graft and prolonged hospitalization for antifungal therapy, experiencing a unique adverse effect.

## Introduction

Pediatric scalp lesions are frequently encountered in the outpatient setting. Common etiologies include seborrheic dermatitis, folliculitis, psoriasis and tinea capitis. However, close follow up is recommended as diagnoses are often clinical. If improvement is not seen, confirming a presumed diagnosis or broadening the differential is necessary, with the aid of subspecialists. Here we present a case of a scalp lesion that worsened over three months despite treatment for presumed tinea capitis with kerion formation. Our patient was found to have an increasingly prevalent fungal infection, multiple superinfections, and required multiple surgeries over six months of hospitalization.

## Case Report

**Fig. 1:**
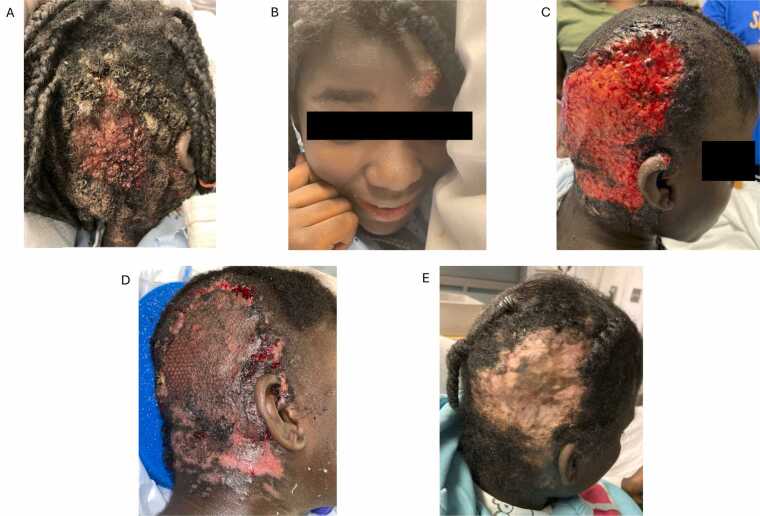
Dermatologic depictions of clinical course. (A) Initial posterior scalp lesion upon presentation to tertiary referral center 3 months after it appeared. (B) Anterior scalp lesion that appeared 2 weeks prior to presentation (C) Posterior scalp lesion at post operative day 2 from initial debridement. (D) Posterior scalp lesion 1-month post operatively from grafting with plastic surgery. (E) Posterior scalp lesion 11 months post-graft with improved skin pigmentation.

A previously healthy 11-year-old African-American female from the Central Valley in California presented initially with a small, raised bump on the right posterior scalp which progressed over 3 months to a large, open, pruritic, painful wound with intermittent bleeding (Fig. 1**A**). Occasionally, the patient noted feeling as though a bug was crawling under the lesion. She had multiple healthcare encounters in the months prior to presentation including to her primary pediatrician, adult dermatology, and several emergency departments (EDs), where she was prescribed cephalexin, clindamycin, and increasing doses of griseofulvin for presumed tinea capitis. Two weeks prior to admission, a new lesion appeared over the contralateral forehead, resembling early stages of the initial lesion (Fig. 1**B**). At home, due to worsening pain and bleeding, the patient's mom shaved her hair and lifted the skin off the affected region and found over twenty “maggots” which she extracted before bringing the patient to the ED. The patient had stable housing, a strong family support system and no prior travel history.

Physical examination on admission was notable for normal vital signs, and a large boggy erythematous eroded plaque with yellow and brown purulent drainage and alopecia on the right parieto-occipital scalp. She also had a 3–4 cm boggy nodule with overlying impetiginized crust on the left forehead.

**Fig. 2:**
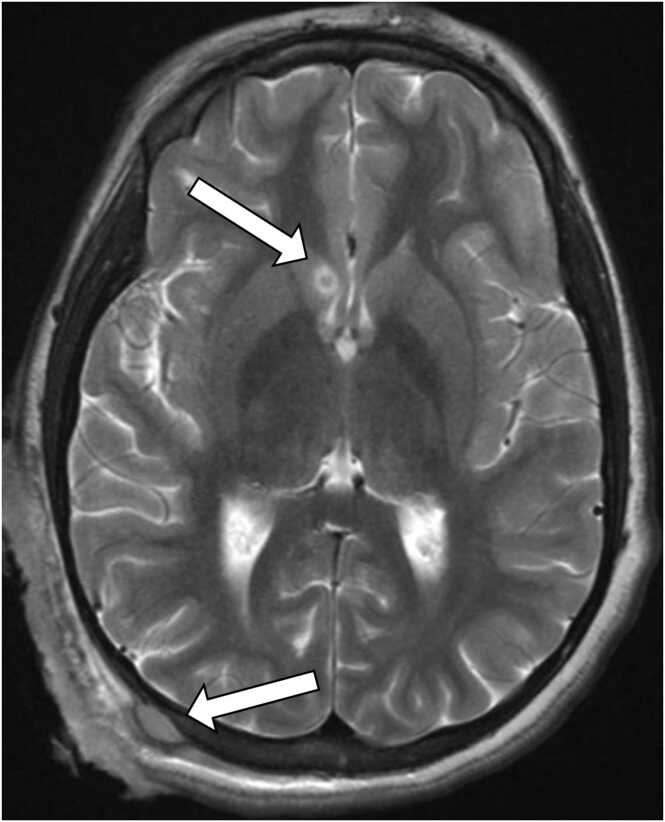
Axial brain MRI (T2) with findings of ulcerative, necrotic mass-like enhancement eroding the right occipital calvaria with epidural extension (bottom arrow); enhancing 4-mm focus around right caudate nucleus (top arrow).

Although most of the maggots were successfully extracted at home, a maggot was noted by nursing on admission but was discarded and not sent for identification. Dermatologic samples obtained on hospital day (HD) 1 for cultures and KOH stain showed evidence of microscopic larval fecal matter, corroborating the history. Empiric vancomycin, ceftriaxone, and fluconazole were initiated. CT and MRI of the brain showed an ulcerative, necrotic mass-like enhancement eroding into the right occipital calvaria with epidural extension and a 4 mm enhancing focus around the right caudate nucleus concerning for septic emboli, suggesting hematogenous dissemination (Fig. 2).

On HD 2, neurosurgery and plastic surgery debrided approximately 280 cm^2^ of scalp skin, subcutaneous tissue, muscle, and bone (Fig. 1C). Per neurosurgical intraoperative examination, a skull defect was debrided but no intracranial intervention was required. Intraoperative wound cultures grew moderate methicillin-sensitive *Staphylococcus aureus, Corynebacterium* species, rare *Acinetobacter* species, and rare *Enterobacter cloacae*. Ceftriaxone was broadened to cefepime, to which all isolates were susceptible; vancomycin was discontinued. On HD 4, multiple intra-operative cultures grew mold, subsequently identified as *Coccidioides spp.* Due to clinical and epidemiologic suspicion, *Coccidioides* complement fixation (CF) had already been sent, resulting positive at 1:128.

To evaluate for dissemination, a CT scan of the chest, abdomen, and pelvis was performed which showed tree-in-bud appearances at bilateral lower lung lobes and mediastinal lymphadenopathy. Ophthalmologic exam found areas of subretinal whitening, potentially representing embolic spread. Cerebrospinal fluid (CSF) fungal, bacterial cultures and *Coccidioides* titers were negative. No other site of osteomyelitis was found outside the skull. Liposomal Amphotericin B (AmBisome) was soon added for improved bone penetration. On HD 21, susceptibilities of fungal cultures revealed a minimum inhibitory concentration (MIC) of 32 mcg/mL to fluconazole, concerning for resistance. Treatment was switched to voriconazole with the added benefit of improved ocular penetration. MICs for voriconazole and amphotericin B were 0.5 mcg/mL and 0.125 mcg/mL, respectively. Pediatric immunology was consulted, and the patient was initiated on IFN-γ therapy, administered as subcutaneous injections with a final dose of 70 mcg thrice weekly for 5 months. She was also on dupilumab during this time per immunology expert opinion given prior success in disseminated coccidioidomycosis [1].

**Fig. 3:**
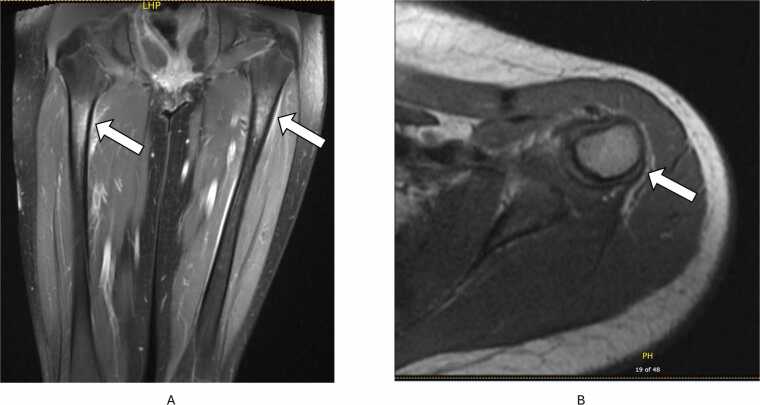
MRI demonstrating areas of marrow edema and periostitis (arrows) correlating with sites of clinical symptoms of pain. After expert review, deemed to be voriconazole-induced periostitis. (A) MRI sagittal view of bilateral femurs with marrow edema in the proximal femoral shafts with associated periostitis. (B) MRI axial view of right humerus with marrow edema and postcontrast enhancement along the medial aspect of the right proximal humeral metadiaphysis.

Given the extensive scalp disease, plastic surgery performed a two stage debridement and skin grafting from the left thigh to the scalp, with satisfactory wound healing and no complications (Figs. 1**C,** 1**D**). With clinical improvement, AmBisome was discontinued after 12 weeks. She remained on voriconazole, however developed new bilateral leg and arm pain one week after discontinuation of AmBisome. MRIs showed multiple areas of periostitis, marrow edema, and enhancement of the left radius, bilateral tibias and femurs (Fig. 3A) raising concerns for new sites of osteomyelitis, despite improving CF titers. AmBisome was resumed, however repeat MRIs two weeks later showed additional changes in the bilateral upper extremities (Fig. 3B). Fluorosis was suspected as a plasma fluoride level returned significantly elevated at 20.8 mcmol/L (normal range <4.1). Further review with musculoskeletal radiologists confirmed the new imaging findings were indeed consistent with fluorosis rather than osteomyelitis. Voriconazole levels during this time ranged from 2.5 to 3.83 μg/mL (therapeutic range 1.00–5.50 μg/mL). The patient was switched to posaconazole with subsequent improvement in imaging and symptoms. Over the hospitalization, the CF titer improved from 1:128 to 1:4 and she was discharged on oral posaconazole after 6 months of admission.

Over a year from her initial presentation, this patient is doing well on daily posaconazole and follows with pediatric infectious disease, plastic surgery, and immunology teams. Her CF titers became negative 10 months after initiation of treatment and have remained negative to date. The patient has not yet been diagnosed with a known clinically significant immunodeficiency. Her scalp graft has healed well but will require ongoing management for alopecia (Fig. 1E).

## Discussion

The steady progression of this case to multiple severe infections highlights the importance of delving into skin lesions more carefully. Initially she was reasonably diagnosed with an atypical presentation of a common infection: tinea capitis with kerion formation and possible bacterial superinfection. However, the multiple healthcare encounters prior to admission without treatment response, and progression to a new contralateral lesion suggested an alternate, atypical etiology. Obtaining confirmatory testing, broadening the differential and subspecialty evaluation is necessary when a patient is not responding to initial empiric treatment. This case highlights the need for primary care clinicians to keep investigating and ensure timely follow-up until a diagnosis is reached.

Coccidioidomycosis presenting as a cutaneous lesion is a rare entity and can be primary or secondary [2]. , In areas where *Coccidioides* is highly endemic, such as California’s Central Valley [3], it should remain on the pediatrician’s differential; as should local endemic fungi in other states. The contribution of race to delayed diagnosis may be considered given the consensus on the lack of medical training focused on Black skin and hair [4]. Lack of access to pediatric subspecialty care in a remote setting may also have delayed diagnosis and will continue to be a barrier in low access areas.

Unfortunately, the untreated *Coccidioides* infection progressed to a severe wound attracting fly larval superinfection. Wound myiasis is a rare clinical presentation in pediatrics and classically associated with housing insecurity, poor hygiene, and tropical regions. It can occur in any tissue or body cavity due to invasion from the larvae of non-biting flies (order Diptera) [5]. Although this case did not have classic predisposing risk factors, the chronicity of the patient’s wound likely contributed to seeding of the lesion. The initial depressed scalp and skull erosion prompted imaging to rule out cerebral myiasis which can be fatal.

*Coccidioides* inoculation typically occurs through inhalation of aerosolized arthroconidia from dry soil and dust. Infection is frequently asymptomatic but can result in pneumonia, responsible for an estimated quarter of community-acquired pneumonia cases in highly endemic areas [3]. Approximately 1 % of cases progress to dissemination, with increased risk in black and Filipino patients [3]. Cutaneous lesions could be primary sites of infection from direct contamination of a traumatic injury with spores [6]. Our patient had pulmonary findings on imaging despite lacking pulmonary symptoms, so it is difficult to discern whether skin or lungs were the primary site of infection. The etiology of the septic embolus found on the initial MRI also remains unclear, with concern for *Coccidioides* meningitis versus bacterial invasion particularly from known *Staphylococcus aureus* superinfecting her wound. *Coccidioides* meningitis was considered less likely given negative CSF studies and the absence of typical imaging such as leptomeningeal enhancement or hydrocephalus, thus she received 6 weeks of antibiotics for possible bacterial septic embolus [3].

Although fluconazole was previously a reliable therapy, resistant *Coccidioides* strains have significantly risen in the past decade [7]. Strong consideration should be given to the use of advanced azoles such as voriconazole, posaconazole or isavuconazole for patients whose disease progresses on fluconazole [7], [8]. Pediatricians should also be aware of common and rare adverse events triggered by prolonged advanced azole therapy. With voriconazole, more well-known adverse effects include gastrointestinal complaints, hepatitis, QT prolongation, visual disturbances, photosensitivity and increased risk for cutaneous cancers. Voriconazole-induced periostitis is a rarely reported complication resembling skeletal fluorosis [9]. This effect is likely from voriconazole’s unique structure as a tri-fluorinated antifungal, which is associated with elevated fluoride levels compared to other azoles [9]. Voriconazole-induced periostitis often presents with diffuse bony pain, swelling, tenderness, and even nodular growths with multifocal involvement. Discoloration of teeth from dental fluorosis may be an initial clue [10]. High dosing, prolonged usage of voriconazole (as in our patient), or decreased renal function may increase risks for complications. Voriconazole discontinuation with transition to another azole is recommended to resolve the periostitis over time [10].

While our patient has shown significant improvement, she still has a long road ahead, including further potential procedures to regain normal hair coverage on the scalp, and psychosocial recovery from prolonged hospitalization. The clinical course demonstrates how multidisciplinary care optimizes diagnoses and a multifaceted approach combining surgical debridement, advanced azoles and possibly immunologic therapy are key to the successful management of severe disseminated coccidioidomycosis.

## Ethical approval

The drafting of this case report including the images used were discussed with the patient and/or their parent/guardian and written consent was obtained and is available on request.

## Contributors statement page

Dr. Duldulao and Dr. Boppana drafted the initial manuscript and revised the manuscript based on critical feedback. Nina Modanlo drafted the abstract of the initial manuscript and contributed to the dermatologic descriptions. Dr. Cheng contributed to the dermatologic writing and pictures. Dr. Malhotra provided supervision through the draft of the initial manuscript. Drs. Malhotra, Nielsen-Saines, Adachi, Kaur, and Dong critically reviewed and revised the manuscript*. All authors approved the final manuscript as submitted and agree to be accountable for all aspects of the work.*

## Funding

Dr. Huan Vinh Dong is supported by the 10.13039/100000002National Institutes of Health (T32 AI177290). No other authors have sources of funding to disclose.

## CRediT authorship contribution statement

**Kristina Adachi:** Writing – review & editing. **Sushma Boppana:** Writing – review & editing, Writing – original draft, Conceptualization. **Nina Modanlo:** Writing – review & editing. **Jared Miguel Duldulao:** Writing – review & editing, Writing – original draft, Project administration, Conceptualization. **Sanchi Malhotra:** Writing – review & editing, Writing – original draft, Supervision, Project administration, Conceptualization. **Carol Cheng:** Writing – review & editing. **Jessica Terrell:** Writing – review & editing. **Karin Nielsen-Saines:** Writing – review & editing. **Ishminder Kaur:** Writing – review & editing. **Huan Vinh Dong:** Writing – review & editing.

## Declaration of Competing Interest

The authors declare that they have no known competing financial interests or personal relationships that could have appeared to influence the work reported in this paper.
